# Formation of Domains within a Lower-to-Higher Symmetry Structural Transition in CrI_3_

**DOI:** 10.1021/acs.inorgchem.3c02970

**Published:** 2023-12-29

**Authors:** Petr Doležal, Marie Kratochvílová, Dávid Hovančík, Václav Holý, Vladimír Sechovský, Jiří Pospíšil

**Affiliations:** Faculty of Mathematics and Physics, Department of Condensed Matter Physics, Charles University, Ke Karlovu 5, 121 16 Prague 2, Czech Republic

## Abstract

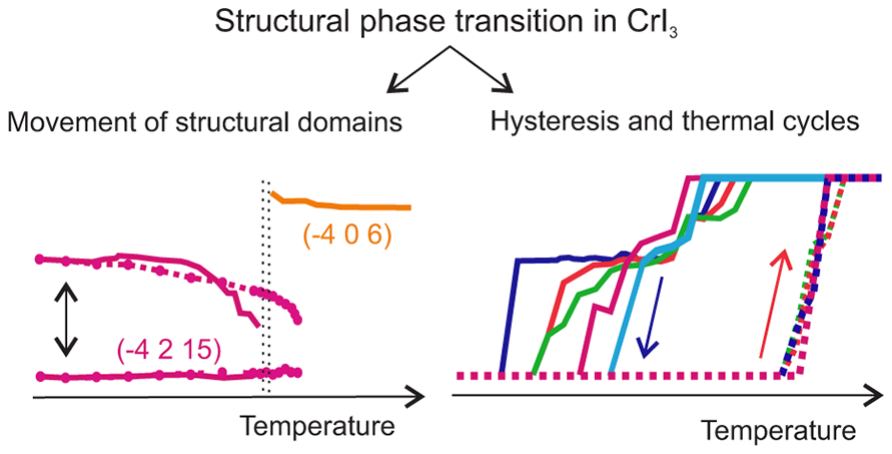

CrI_3_ represents one of the most important van der Waals systems on the route to understanding 2D magnetic phenomena. Being arranged in a specific layered structure, it also provides a unique opportunity to investigate structural transformations in dimension-confined systems. CrI_3_ is dimorphic and possesses a higher symmetry low-temperature phase, which is quite uncommon. It contrasts with vanadium trihalides, which show a higher symmetry high-temperature phase. An explanation of this distinct behavior, together with a large cycle-dependent transition hysteresis, is still an open question. Our low-temperature X-ray diffraction study conducted on CrI_3_ single crystals complemented by magnetization and specific heat measurements was focused mainly on specific features of the structural transition during cooling. Our results manifest that the structural transition during cooling relates to the formation of structural domains despite the lower symmetry structure transforming to a higher symmetry one. We propose that these domains could control the size of thermal hysteresis.

## Abstract 2

Findings in this study show that structural phase transitions in CrI_3_ have a large hysteresis, which depends on temperature cycles. It is connected with the formation of new mosaic blocks during the structural transition, which is quite surprising because the transformation is from low to higher structural symmetry.

## 1 Introduction

The CrI_3_ compound belongs to magnetic van der Waals (vdW) materials which have been intensively studied in recent years mainly for possible applications in spintronics and optoelectronics^1−3^ and also as objects suitable for testing two-dimensional (2D) magnetic toy models.^4^ The 2D character of transition metal trihalides TX_3_ (T—transition metal, X—halide) allows the preparation of thin layers needed for engineering applications. Chromium trihalides CrI_3_, CrBr_3_, and CrCl_3_ are among the pioneering materials in 2D magnetism research. CrI_3_ and CrBr_3_ are ferromagnetic (FM) with *T*_c_ = 61^5,6^ and 37 K,^7^ respectively, whereas CrCl_3_ shows antiferromagnetic (AFM) order below *T*_N_ = 17 K.^8,9^ Monolayers of all three compounds were reported to be FMs.^10−12^ The vanadium counterparts are FM (VI_3_^13,14^) and AFM (VBr_3_^15,16^ and VCl_3_^17^).

The high-temperature (HT) structure of the three VX_3_ compounds is the rhombohedral one (*R*-3) which undergoes a structural transition with cooling through the transition temperature *T*_s_ (which lies between 79 and 100 K for all three VX_3_ compounds^17^). The low temperature phases are not properly resolved yet. On the contrary, the HT phase of CrX_3_ compounds is monoclinic AlCl_3_-type (*C*2/*m*),^13,14^ and cooling leads to a structural transition connected with increasing symmetry from monoclinic to rhombohedral (space group *R*-3),^18,19^ which is unusual in solid-state physics. For an overview of structural transition sequences in CrI_3_ and VX_3_ halides, see Figure S1.^20^ This structural transformation exhibits a large thermal hysteresis (more than 40 K), which is in strong contrast with the negligible hysteresis of the structural transitions in VI_3_ and VBr_3_.^17,21^ Moreover, the size of transition hysteresis in CrI_3_ is thermal history-dependent, in particular, it depends on the number of cooling–warming cycles over the transition as reported by McGuire et al.^5,18^ Interestingly, Niu et al. have recently discovered that stacking of vdW layers on the surface of CrI_3_ flakes in the low-temperature (LT) phase corresponds to the bulk monoclinic structure of the HT phase and the rest of the inner layers keeps the bulk stacking order typical for the rhombohedral structure.^22^ A recent synchrotron X-ray powder diffraction study on bulk CrI_3_ samples revealed the coexistence of both monoclinic and rhombohedral crystal structures down to 10 K.^23^

The phase of the LT structure is usually less symmetric, which implies the formation of domains in the sample and consequently affects the lattice parameters. However, the reversed symmetry order of the LT and HT phases observed in CrI_3_ raises a fundamental thermodynamic question regarding domain formation, the type of domains, and the transition mechanism itself. It is of high importance to understand this process as the magnetic properties are closely related to the stacking order of the vdW layers, especially in CrI_3_.^24^ The theoretical studies have shown that the monoclinic stacking in CrI_3_ favors an antiferromagnetic ground state, whereas the rhombohedral stacking results in ferromagnetic order.^22,24^ These findings point to the importance of studying structural transformation for understanding magnetic properties.

In this work, we studied the peculiarities of the structural transition in CrI_3_ by X-ray single-crystal diffraction, magnetization, and specific heat measurements. The study revealed the formation of unusual structural domains during the transition upon cooling and evidenced the relation to magnetic and thermal properties.

## 2 Experimental Methods

CrI_3_ single crystals of various sizes have been grown from pure elements (Cr 99.99% and I_2_ 99.999% both permanently stored inside a glovebox under an Ar inert environment) using the chemical vapor transport method. First, a quartz tube with Cr metal powder was kept at 300 °C and evacuated overnight down to a vacuum of 10^–7^ mbar for proper degassing. Then, the tube was filled with 6 N argon gas, and a 2% over stoichiometric amount of I_2_ was added. Then, the tube was pre-evacuated by a Scroll pump and evacuated by a turbomolecular pump for 5 min. Finally, the sealed quartz tube was inserted into a gradient furnace and slowly heated by 0.2 K/min to react Cr and I_2_. Finally, a thermal gradient of 650/500 °C was kept for 4 weeks to grow CrI_3_ single crystals. The single crystals of black-reflective color of several millimeter square dimensions have been obtained. The desired 1:3 composition was confirmed by EDX analysis. Our process reproduces the process described by McGuire et al.^5^ The black reflective single crystals in the form of flat flakes of several millimeter sizes were obtained (see Figure S2).^20^ The LT X-ray diffraction was performed on the refurbished Siemens D500 θ–θ diffractometer in the Bragg–Brentano geometry using the Cu_Kα1,2_ radiation. The He closed cycle was used for cooling. The temperature was controlled by the LT cryostat (*ColdEdge*), with temperature stabilization better than 0.1 K and with an absolute uncertainty of 0.5 K in comparison to the PPMS cryostat (*Quantum Design, Inc.*). The sample chamber was filled with He gas to ensure good thermal contact between the sample and the cold finger. A piezo-driven rotator was used for alignment of the sample in the ϕ-direction. This experimental setup allows measurements in the temperature range of 3–300 K. The reciprocal space maps were measured by a position-sensitive detector Mythen 1K. The limitations in sample alignment allow the measurement of only one diffraction maximum per temperature cycle. The CrI_3_ samples are partially sensitive to moisture, and this is also the reason why different single crystalline samples from the same batch were used for the specific heat, magnetization, and diffraction measurements. All these samples were plates and had comparable sizes around 1 × 3 mm^2^. The presented X-ray diffraction study consists of two different single crystalline samples. The first one was used for the determination of lattice parameters and the study of the behavior of domains during cooling. The second one was used for the study of temperature hysteresis using the (0 0 24) maps. All samples were almost identical plates with a size of around 1 × 0.5 mm^2^ in both cases, and the whole sample surface was irradiated by an X-ray beam. The magnetization curves were measured by an MPMS-7 SQUID magnetometer (*Quantum Design, Inc.*). The heat capacity data were measured by a PPMS9T (*Quantum Design, Inc.*) using the heat-pulse method as the standard relaxation method principle is not sensitive to first-order phase transitions. It has to be noted that there are no uncommon significant hazards or risks associated with this work.

## 3 Results and Discussion

**Figure 1:**
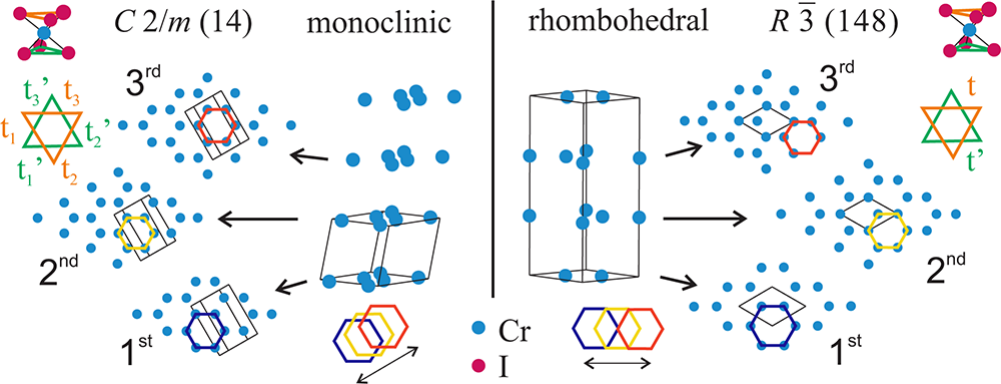
Comparison of monoclinic and rhombohedral crystal structures of CrI_3_. The colored triangles represent distortion of a regular octahedron. The colored hexagons show the different shifts of layers in the monoclinic and rhombohedral crystal structures. It has to be noted that the 4th layer overlaps exactly with the 1st layer (perpendicular view on the basal plane) in the rhombohedral structure. On the other hand, in the monoclinic structure, there is no such direct overlap, but the shift between the 1st and 4th layers is very small.

To understand the difference between monoclinic and rhombohedral stacking, we compare the LT and HT structures in more detail. The unit cells of both crystal structures are shown in Figure 1 (for simplicity, only the Cr atoms are shown). Note that for the description of the rhombohedral structure model, we use the conventional hexagonal unit (e.g., the label *c*_hex_ refers to the *c* lattice parameter of the hexagonal unit cell in the rhombohedral phase). The monoclinic structure contains only one Cr layer per unit cell. On the other hand, the rhombohedral structure has three such layers resulting in *c*_hex_ ∼ 3*c*_mono_. The Cr atoms are ordered as hexagons within the layers. The hexagons are regular in a rhombohedral structure and slightly distorted in the monoclinic phase in which each layer is offset relative to the adjacent Cr layers. This shift is the most pronounced difference between the HT and LT structures (see the colored hexagons in Figure 1). In the monoclinic phase, the layers are shifted in the direction of the hexagon vertex, while in the rhombohedral structure, the layers are shifted in the direction perpendicular to the side of the hexagon. The vdW interaction between the layers is weak in comparison to the covalent bonds between Cr and I atoms within the layers, which probably controls the crystal structure. One Cr atom is surrounded by 6 neighboring I atoms, creating an octahedron. In both crystal structures, the octahedron is not regular (see the corners of Figure 1). In the rhombohedral structure, it consists of two different equilateral triangles, while in the monoclinic crystal structure, the triangles are scalene. A direct comparison of the crystal structures is not straightforward because no t-group–subgroup relation allows the description of a more symmetrical structure in the t-subgroup. However, some similarities can be inferred; *c*_hex_ ≈ 3*c*_mono,_*a*_hex_ ≈ *a*_mono,_*b*_mono_ ≈ *a*_hex_ giving us a hint of changes during the structural transition. The diffraction maxima (0 0 *l*) in a symmetrical direction should be only shifted, and the appearance of new ones is not expected because of extinction rules in the rhombohedral space group. This behavior was confirmed by the measurement of a symmetrical θ–2θ scan (see Figure S3 in the Supporting Information^20^). Since the structural transition is from a lower symmetry to a higher symmetry crystal structure, no 2θ splitting of diffraction maxima connected with the creation of domains by the twinning mechanism should be observed. Our results also show that the full width at half-maximum of diffraction 2θ profiles is comparable in both phases and slightly bigger for the rhombohedral phase (see Figure S4(20)).

**Figure 2:**
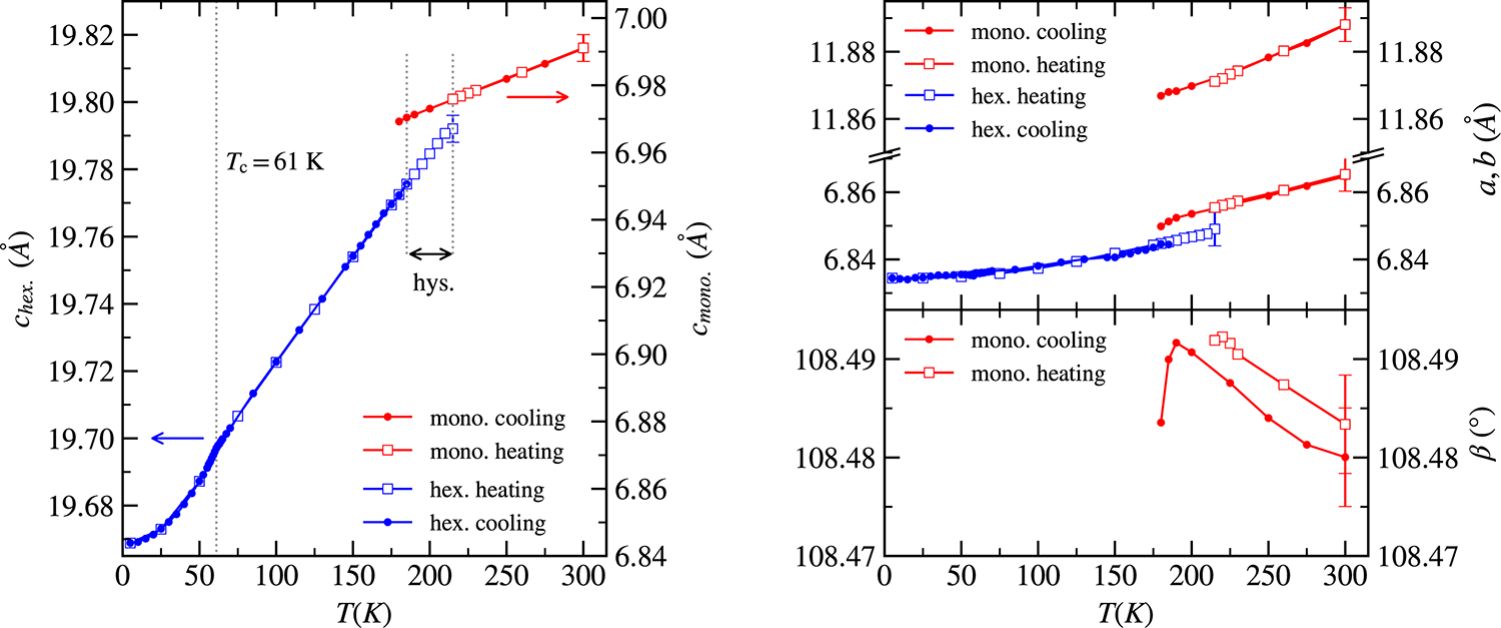
Temperature dependence of the lattice parameters in monoclinic and rhombohedral crystal structures. The hexagonal unit cell is used for the rhombohedral crystal structure, and the corresponding lattice parameters are labeled by subscript hex. The experimental error connected with the precision is smaller than the size of the data point. The error bars in the plots show the error connected with the accuracy.

**Figure 3:**
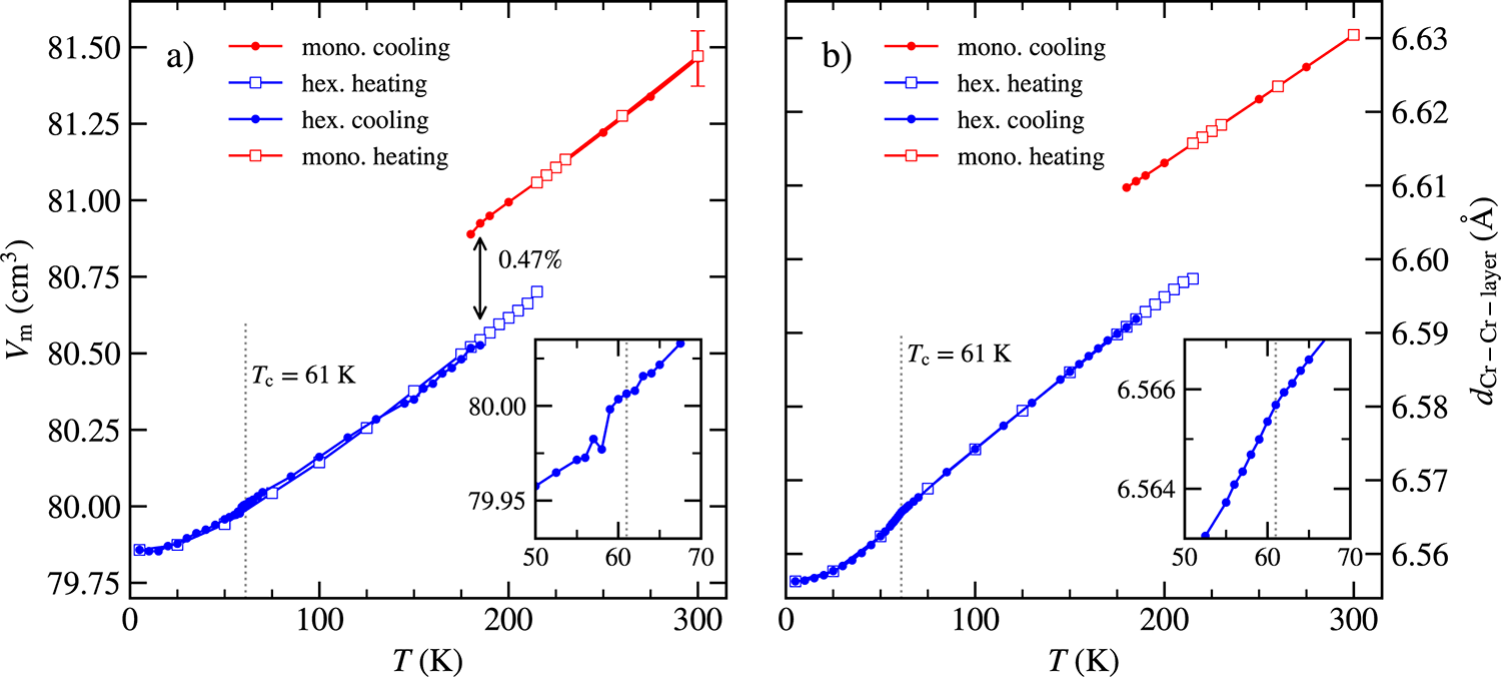
(a) Molar volume of monoclinic and hexagonal crystal structures as a function of temperature. The inset shows behavior close to the ferromagnetic transition. (b) Temperature dependence of distance between Cr layers, which is proportional to the vdW gap. The experimental error connected with the precision is smaller than the size of the data point. The error bar in panel (a) shows the error connected with the accuracy.

The reciprocal space maps of (0 0 8)_mono_, (0 6 6)_mono_, (−4 0 6)_mono_, and (4 0 6)_mono_ diffraction peaks were measured to determine the lattice parameters as a function of temperature. The maps were integrated in a rocking direction to obtain 2θ profiles which were fited by the pseudo Voigt function. The lattice parameters were determined from these 2θ positions. It is important to emphasize that a plate-like sample’s shape (c*_mono_ is perpendicular to this plate) limits the number of diffraction maxima that are accessible for measuring in our configuration (for more details about alignment, see Section II). The above-listed monoclinic diffraction maxima cannot be related to the rhombohedral ones detected below the transition temperature; however, close to their positions in 2θ, we measured diffraction peaks (0 0 24)_hex_, (0 3 18)_hex_, (−4 2 15)_hex_/ (4 −2 15)_hex_, and (4 −2 21)_hex_/ (−4 2 21)_hex_. The resulting lattice parameters are shown in Figure 2. The dominant thermal contraction is observed for the *c*_mono_ and *c*_hex_ lattice parameters. The ferromagnetic ordering at 61 K relates to the change in the slope of the *c*_hex_ lattice parameter. No such anomaly is observed in the basal plane in the *a*_hex_. The β-angle in the monoclinic structure exhibits only a small change of 0.012° during cooling. To compare the volumes of monoclinic and rhombohedral phases, the molar volume is plotted in Figure 3a) (comparing the volume of unit cells would be misleading since there is no group–subgroup relation). During the transition, the volume was reduced by almost 0.5%, which is quite a huge change in comparison to the vanadium-based vdW compounds like VBr_3_ (0.08%)^17^ and VI_3_^21^ where the change of volume was negligible. The change in volume during the ferromagnetic transition is on the border of experimental error.

The vdW gap is directly related to the distance between Cr layers *d*_Cr–Cr-layer_ and not to the *c* lattice parameter since in the monoclinic structure ***c*** is not perpendicular to the ***ab*** plane. The *d*_Cr–Cr-layer_ dependence is shown in Figure 3b. In both crystal structures, the vdW gap decreases with decreasing temperature, with a higher slope in the rhombohedral phase. All diffraction maxima were measured during cooling and heating to test whether the change in lattice parameters was reversible. The overlap of heating and cooling curves (see full and open symbols in Figures 2, 3, and S5(20)) demonstrates no effect of temperature cycling on lattice parameter values.

**Figure 4:**
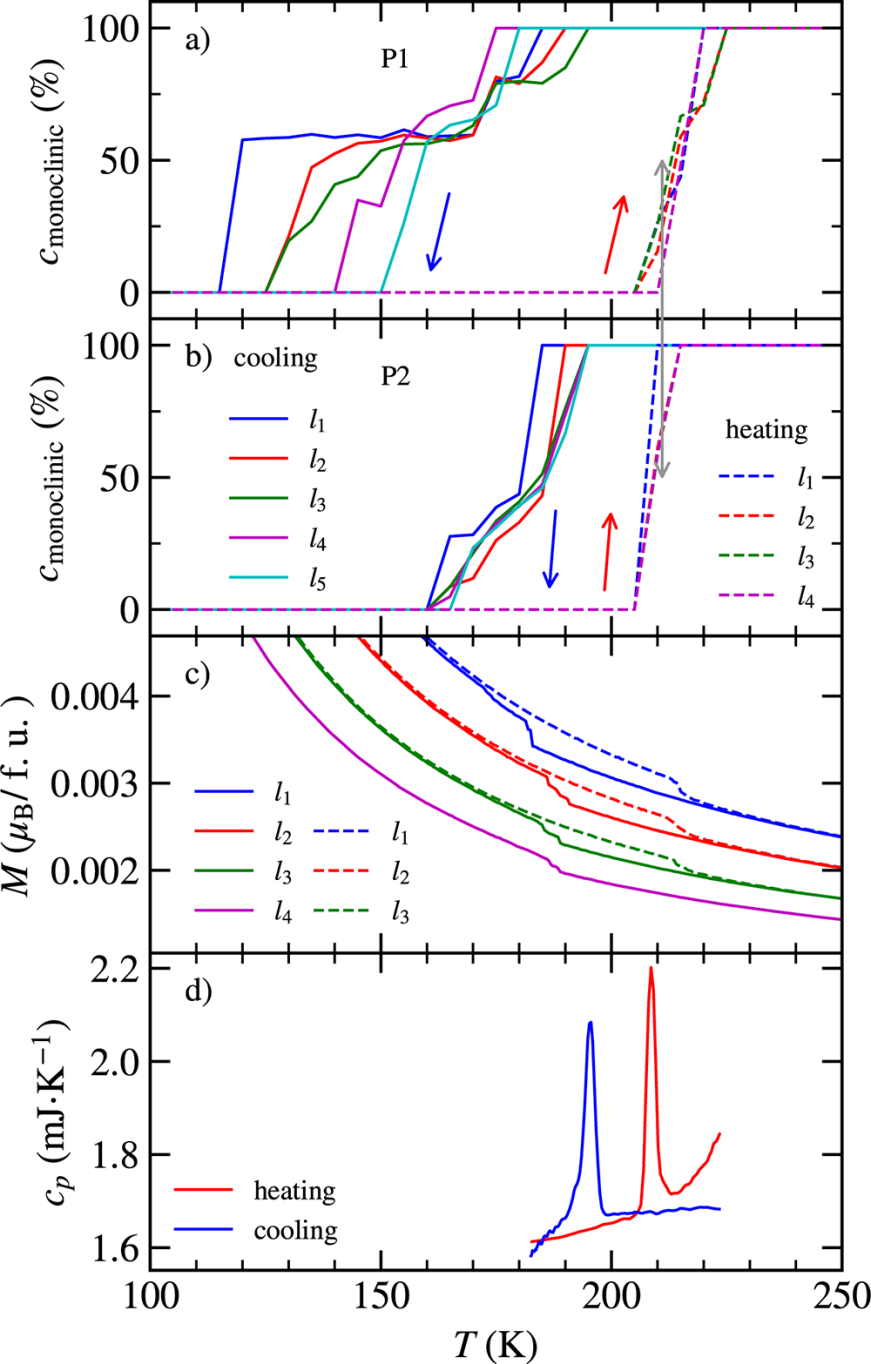
(a, b) Portion of the monoclinic phase in the sample as a function of temperature. *l*_*i*_ refers to the number of temperature cycles (one cycle represents the cooling and heating curve). P1 and P2 show the behavior of contributions having different ω_offset_. (c) Change of hysteresis in magnetization curves during the structural transition. (d) Specific heat of the CrI_3_ single crystal measured by the single-pulse method. The area below the peaks can serve as an estimation of the difference between latent heat during cooling and heating. This difference is only 1.6%. The samples used for diffraction, magnetization, and specific heat measurement were different.

A large temperature interval of phase coexistence during the structural transition was already observed in previous studies, where the dependence of its size on thermal cycles was reported.^5^ In our experimental setup, only one diffraction maximum can be measured during the cooling and heating cycles. Therefore, we tested the thermal robustness of the hysteresis by choosing (0 0 8)_mono_ and (0 0 24)_hex_ diffraction peaks measured on a single crystalline sample without a cooling history. We used the integral intensity of the peaks to determine the ratio of the monoclinic and rhombohedral phases within the coexistence interval. Figure 4a,b displays the monoclinic volume fraction as a function of temperature for five temperature cycles. It has to be noted that the diffraction maps of (0 0 8)_mono_ contain two separated mosaic blocks referred to as part 1 (P1) and part 2 (P2). Each part has a quite large mosaicity up to 2.5° (see Figures S6 and S7(20)).

We do not think that this mosaicity comes from sample manipulation before the measurement (previous strain stress experience). The sample was placed only on a horizontal glass plate without fixed mounting or gluing. We tried to manipulate the sample as little as possible. The mosaic structure of the sample is intrinsic and, most likely, developed during the sample growth by movement of dislocations, creating small-angle boundaries between the mosaic blocks. However, this effect should be investigated in more detail. Unfortunately, previous structural studies did not show the ω_offset_-profiles, which would allow the direct comparison and also direct comparison of quality between single crystals. To our knowledge, our study is the first in this sense.

Despite having identical lattice parameters, P1 and P2 exhibit entirely different hysteresis behavior (see Figure 4a,b). During cooling, the transition in P1 happens in two steps. The fractional volume of 40% transforms to the rhombohedral phase at ≈175 K and the rest of 60% at a substantially lower temperature of ≈117 K. During heating, the transition has a character of a sharp step at 215 K. The behavior of P2 during cooling is different because the lowest transition happens around 165 K (see Figure 4b). This phenomenon can be understood from the diffraction map, where we can see the ω_offset_-dependence (Figure S7).^20^ The ω_offest_ is an angle between the diffraction vector and normal vector of the sample surface (see Figure S8(23)). We can observe that each transition in P1 is connected with a different domain in the sample (see Figure S7(20)). P1 is a sum of mosaic blocks with large (≈96 K) and small (≈40 K) hysteresis. As Figure 4a shows, the cooling/heating cycling gradually shrinks the large hysteresis of P1 in an asymmetric way, i.e., by shifting the lower transition temperature up during cooling, while the transition upon heating remains almost intact. After the fifth cycle, the large and small hysteresis in P1 is almost identical and comparable to the hysteresis of P2. Similar evolution, depending on temperature cycling, displays the magnetization curve in Figure 4c.

This observation in the previous paragraph raises a question of interpretation of the difference between domains with large and small hysteresis. The chemical composition can be excluded as the lattice parameters are identical for both domains. Presumably, mechanical properties such as grain size, the number of defects, etc., might be responsible for the effect. However, that would imply that the transition is connected with the formation of defects or new domains in the rhombohedral phase. Especially, the creation of new domain groups would be quite unconventional assuming that the transition is from a structure of lower symmetry to the one of higher symmetry with no twinning as is usually the case of a symmetrically reversal transition.

**Figure 5:**
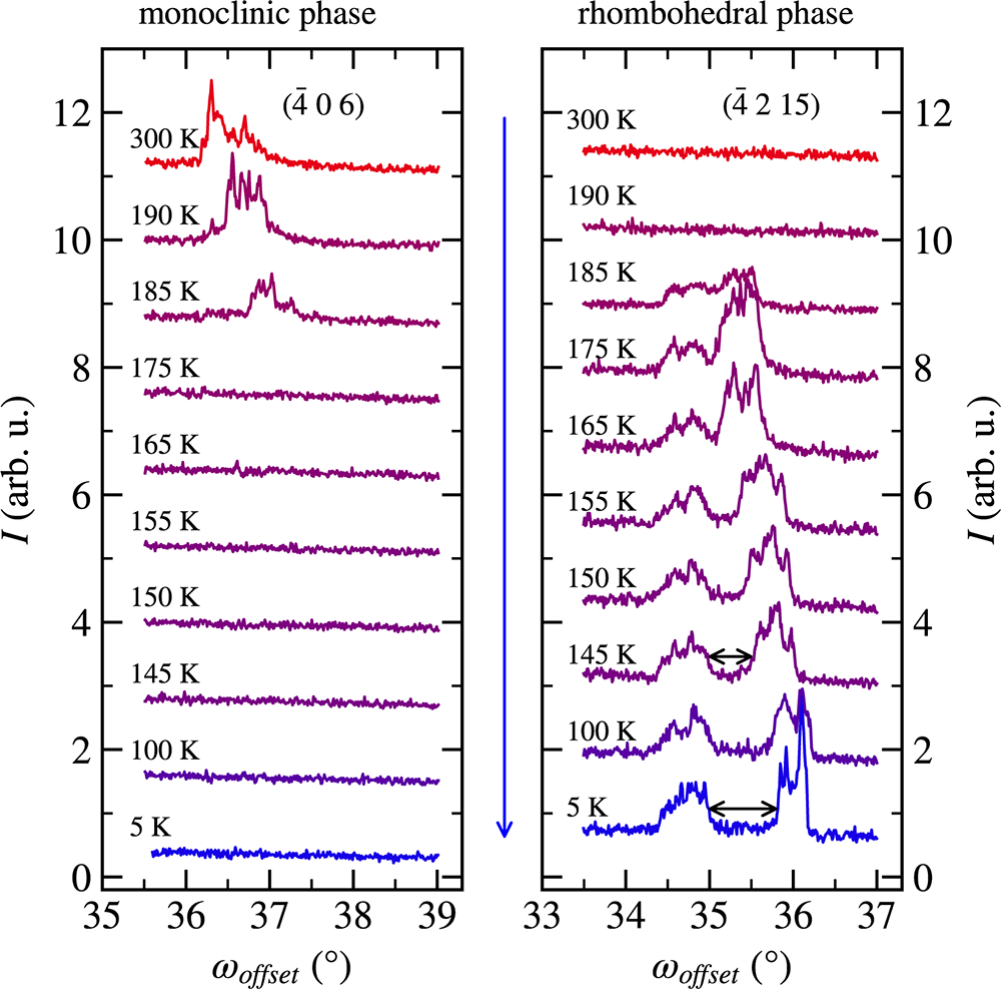
ω_offset_ profiles of (−4 2 15)_hex_ and (−4 0 6)_mono_ diffraction maxima. Two different contributions and their separation during cooling are visible in the first panel. The blue arrow indicates the cooling cycle. The curves are shifted along the *y*-direction for better readability.

To get a deeper picture of what happens with the domains during the structural transition, we investigated the ω_offest_ profiles of the (−4 0 6)_mono_ and (−4 2 15)_hex_ diffraction peaks. The (−4 0 6)_mono_ diffraction peak is in the *a*_mono_*c*_mono_ plane (see Figure S9(20)). We emphasize that ω_offest_ is very sensitive to the sample’s tilt, which is changing during cooling and heating. Therefore, the direct comparison of peak shapes and their intensities could be misleading, and only qualitative information about the behavior of the whole ω_offset_ profile is relevant. In the monoclinic phase, we can see a shift of the whole ω_offset_ profile shown in Figure 5, which is a result mainly of the lattice parameter change and tilt of the sample during cooling. In the rhombohedral phase right after the phase transition, the ω_offset_ profile for (−4 2 15)_hex_ comprises two main components. These components start to separate from each other during further cooling (Figure 5).

**Figure 6:**
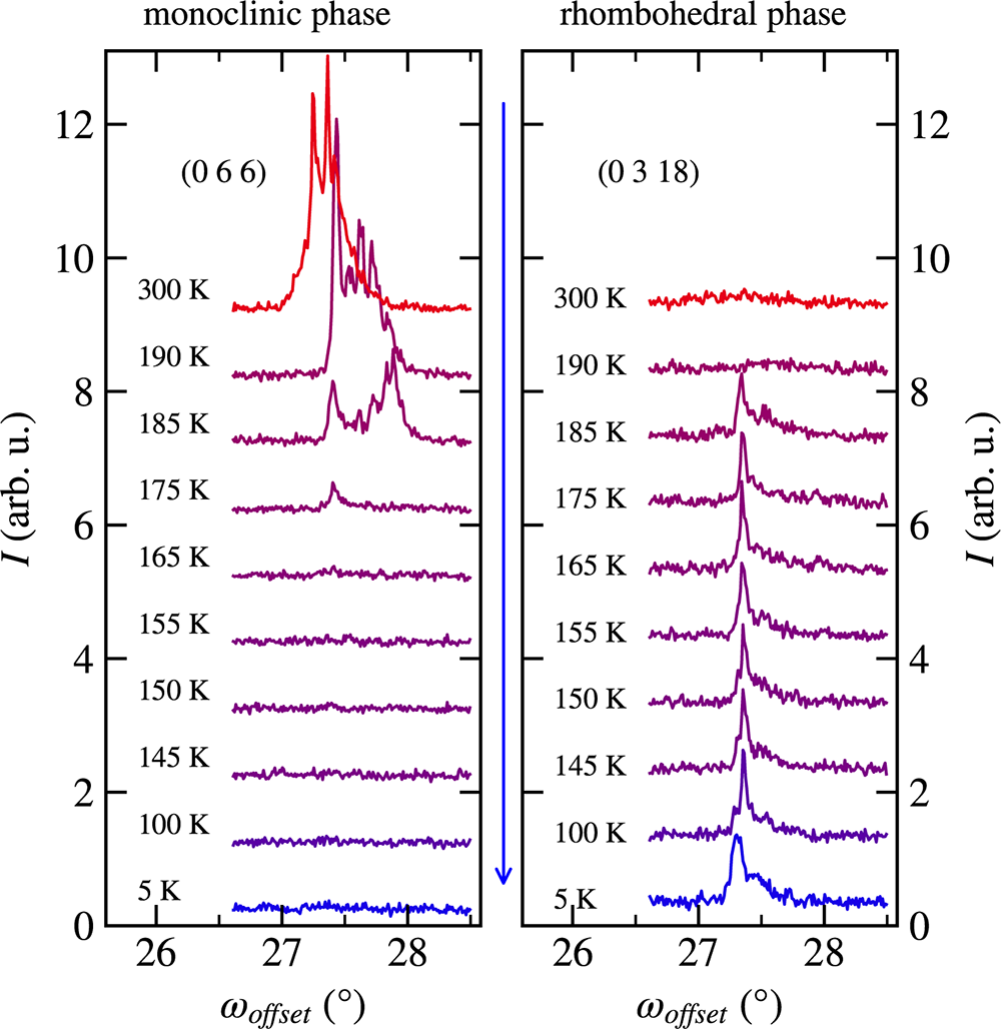
ω_offset_ profiles of (0 3 18)_hex_ and (0 6 6)_mono_ diffraction maxima. Only one contribution is visible in the rhombohedral and monoclinic phases during cooling. The blue arrow indicates the cooling cycle. The curves are shifted along the *y*-direction for better readability.

**Figure 7:**
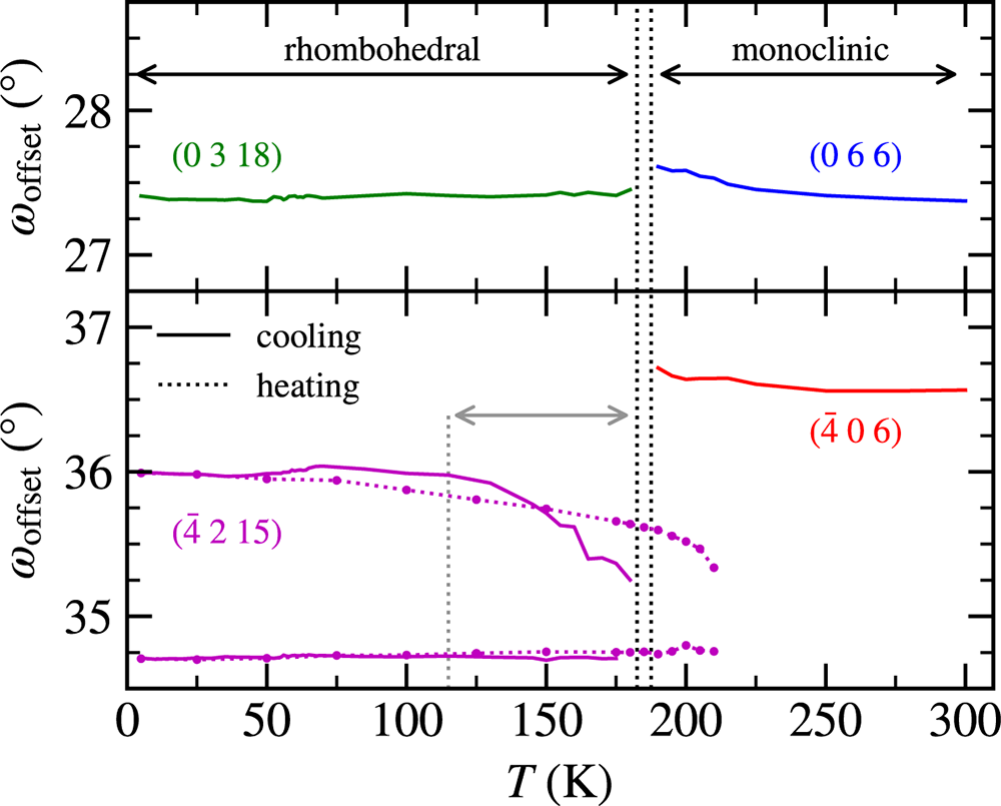
Comparison of the temperature dependence of the ω_offset_ profile of (0 6 6)_mono_, (−4 0 6)_mono_, (−4 2 15)_hex_, and (0 3 18)_hex_ diffraction maxima. The gray arrow indicates a temperature interval of 115–185 K with the biggest movement of domains. The narrow interval between dotted black lines corresponds to the coexistence of both phases during the transition at the cooling cycle.

On the other hand, no such splitting was observed in the case of (0 6 6)_mono_/(0 3 18)_hex_ diffraction (see Figure 6). Figure 7 shows the temperature dependencies of these ω_offset_ profiles. One component of (−4 2 15)_hex_ is almost temperature-independent, while the second component rapidly increases its ω_offset_ between 180 and 115 K and saturates below 100 K. This observation corroborates a scenario of the domains’ structure re/formation during the transition and their thermally induced movement. During heating, the movement of the second component is opposite, returning to the starting ω_offset_ position (see Figure 7). Interestingly, the transition to the HT phase takes place exactly at the temperature at which the ω_offset_ separation of the components is approximately the same as at the transition temperature during cooling. This demonstrates a direct impact of domains on the hysteresis of structural transition and explains the possibility of having domains in the sample with different hysteresis behaviors as we observed in samples without cooling history (see Figure 4). The formation of a new domain block was observed not only in this sample but also in another sample which is not used for data analysis (see Figure S10).

To point out the differences, we compare the structural transition in CrI_3_ with VX_3_. In these compounds, the LT phase has lower symmetry than the HT phase.^13,17,21^ Therefore, the structural transition results in the formation of domains; however, these domains have different origins, i.e., they originate from a lattice distortion. Hence, the temperature dependence of lattice parameters *a* and *b* is opposite, similar to a transition from austenite to martensite.^25^ In contrast, the domains in CrI_3_ are formed without distortion of the lattice. As a result, the structural transition is much more sensitive to the real structure of the sample and its mosaicity, whereas in VI_3_ and VBr_3_, the domain structure is established in each mosaic block separately; hence, the transition is less sensitive to the original domain/mosaic structure. Our results can also explain why the monoclinic and rhombohedral phases coexist in CrI_3_ below 10 K in the powder X-ray diffraction study reported by Maseguer-Sánchez et al.^23^ In their data, the transition is broadened, and at 10 K, 10% of the sample is still in the monoclinic phase. The structural transition then looks unfinished. The explanation might originate in the mechanical treatment to get a powder sample since, revealed by our observation, the transition is very sensitive to structural defects and grain boundaries.

CrCl_3_ exhibits very similar behavior showing cycle-dependent hysteresis and having comparable temperatures of the structural transition,^19^ suggesting that a similar mechanism like in CrI_3_ can be expected. In CrBr_3_, the structural transition at 423 K is known from the literature^26^ and has not been studied in detail yet. The comparison of these two FM counterparts highlights the uniqueness of the structural transition of CrI_3_. The residual monoclinic stacking in bulk CrI_3_ single crystals^23^ is projected to LT magnetization data by a small antiferromagnetic contribution^27^ which was not detected in bulk CrBr_3_^28^ although antiferromagnetic order was proved by magnetoconductance measurements on CrBr_3_ tunnel barriers realized with multilayer-exfoliated samples with various stacking arrangements^29^ (see also the comparison of 2 K magnetization data on our CrI_3_ and CrBr_3_ single crystals in Figure S11).

## 4 Conclusions

We studied the structural transition in the vdW compound CrI_3_ by X-ray single-crystal diffraction, magnetization, and specific heat measurements and discussed the results in comparison with that in vanadium trihalides. We determined the lattice parameters as a function of temperature in the range of 3–300 K and specified the change of the volume during the structural transition. Our study revealed the formation of new domain groups within cooling CrI_3_ when the lower symmetry (monoclinic) structure transforms into a more symmetric rhombohedral structure, contrary to a standard martensitic transformation observed in vanadium trihalides. In this case, the domains cannot form due to a distortion of the crystal lattice within the transition. During cooling, the transition temperature strongly depends on the thermal history of the sample (115–185 K), whereas the transition temperature during heating remains intact (around 215 K) by any change in thermal history. In our understanding of the complex behavior of CrI_3_, the lattice defects and probably the domain structure affect the magnitude of the thermal hysteresis of the structural transition. The transition temperature is primarily determined by thermodynamics, and the role of the domain structure is unclear.

## Supporting Information Available

Temperature dependence of the θ–2θ scan, comparison of 2θ profiles in monoclinic and hexagonal phases, temperature dependence of (0 0 24)_hex_ diffraction maxima, plot of ω_offset_ profiles and corresponding ω_offset_–2θ maps, graphical representation of diffraction angles, and crystallographic lattice planes within the unit cell using the VESTA software^30^ (PDF)
