# Supplementary material for: Formation of Domains within a Lower-to-Higher Symmetry Structural Transition in CrI3
Source: Inorg Chem. 2023 Dec 29;63(2):976–82. doi: 10.1021/acs.inorgchem.3c02970 (PMC10792599; doi:10.1021/acs.inorgchem.3c02970)
Supplement: Supplementary file 1 — ic3c02970_si_001.pdf [file ic3c02970_si_001.pdf]

Supporting Information for:

Formation of domains within lower-to-higher symmetry structural transition in CrI_3

Petr Doležal[1,*], Marie Kratochvílová[1], Dávid Hovančík[1], Václav Holý[1], Vladimír Sechovský[1], Jiří Pospíšil[1]

[1]*Charles University, Faculty of Mathematics and Physics, Department of Condensed Matter Physics, Ke Karlovu 5, 121 16 Prague 2, Czech Republic*

* *Corresponding author's email address: petr.dolezal@matfyz.cuni.cz*

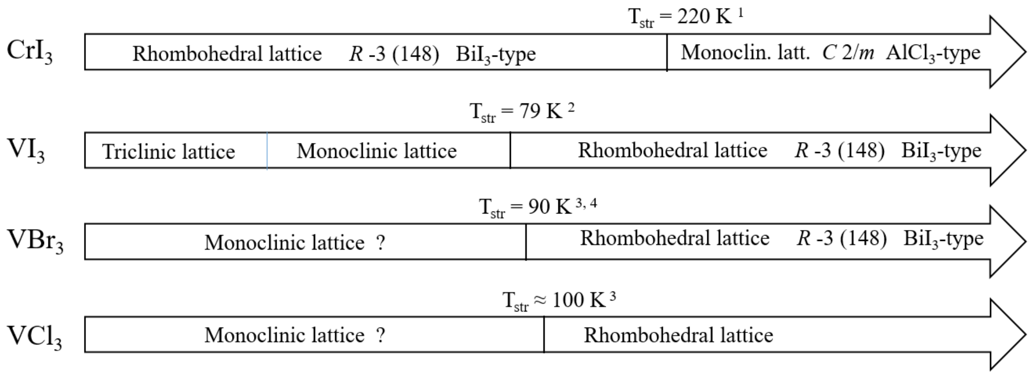

Fig. S1: Schematic overview of crystal structures and lattices of vdW halides. The missing space groups of structural phases of VX_3 were not so far successfully resolved.

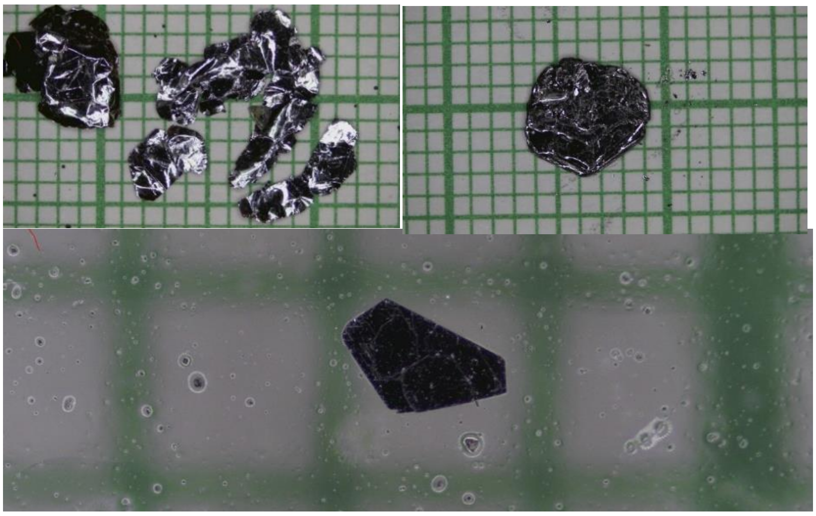

Fig. S2: Representative examples of CrI_3 single crystals produced by the chemical vapor transport method. The size and shape of the single crystals were from large bulk pieces to submillimeter large tens-micron thin plates. Small samples with clear hexagon edges were primarily selected for the measurements. The bottom picture shows the sample, which was used for the measurements and results presented in our work.

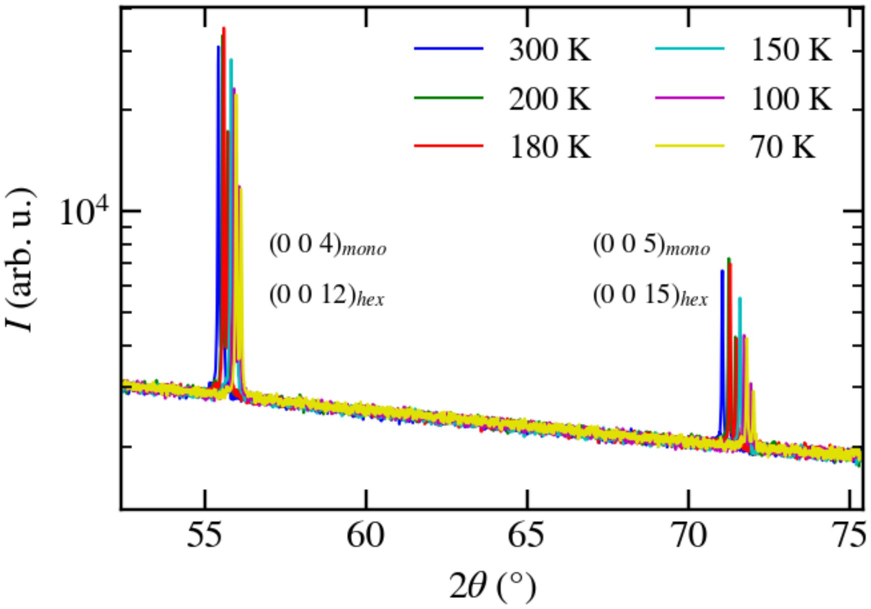

Fig. S3: $\theta - 2\theta$ scans between (0 0 12)$_{hex}$ and (0 0 15)$_{hex}$ diffraction maxima showing that there is no other intensity between them, which would be a sign of disorder in c_{hex} direction. It has to be noted that the $Cu_{K\alpha 1,2}$ radiation was used and the diffraction peaks are doublets.

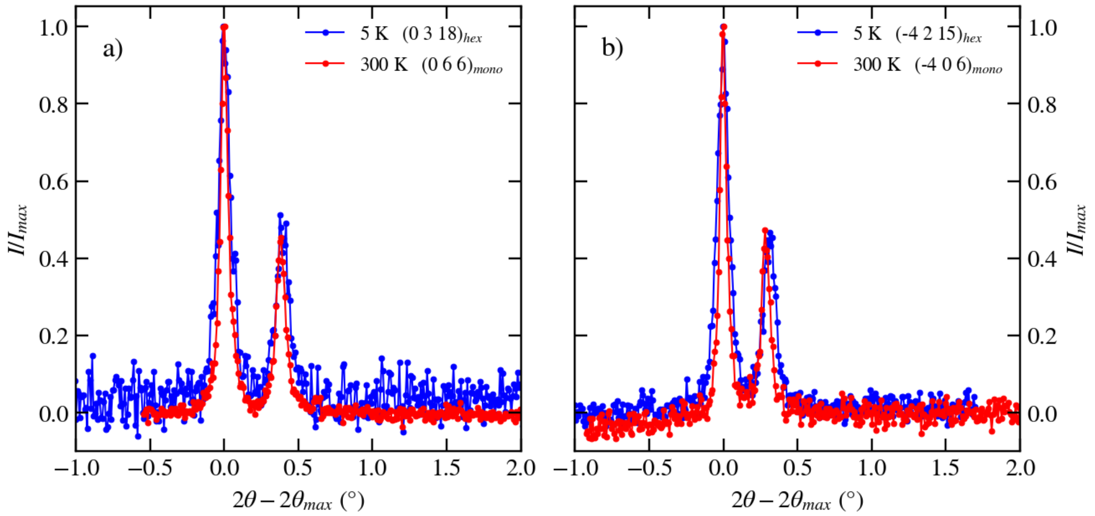

Fig. S4: Comparison of 2θ profiles in the monoclinic and rhombohedral phases. The hexagonal and monoclinic peaks are on almost the same 2θ angle and therefore they could be compared. In general, a slightly bigger full width at half maximum is observed for the rhombohedral phase. It has to be noted that the $Cu_{K\alpha 1,2}$ radiation was used and therefore the diffraction peaks are doublets in panels a) and b).

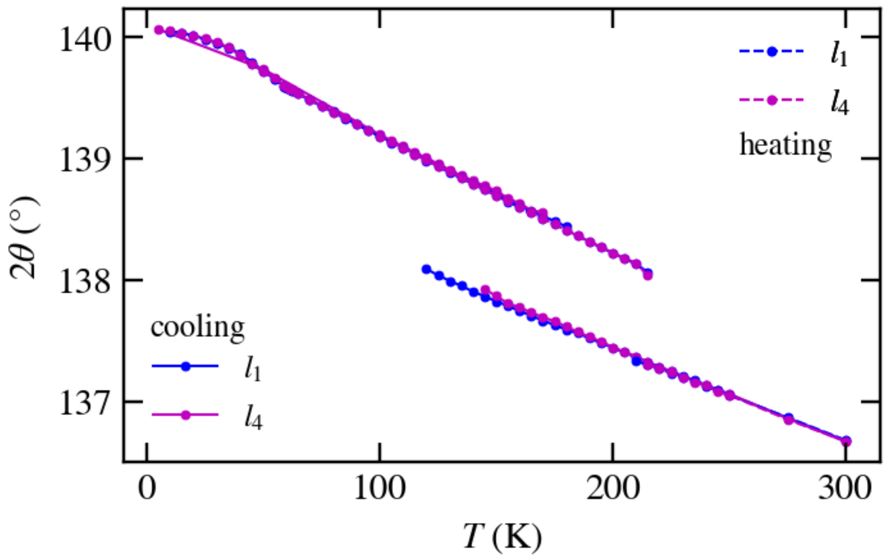

Fig. S5: Temperature dependence of $(0\ 0\ 24)_{hex}$ diffraction maxima. The overlap of l_1 and l_4 data points shows the independence of lattice parameters on temperature cycles.

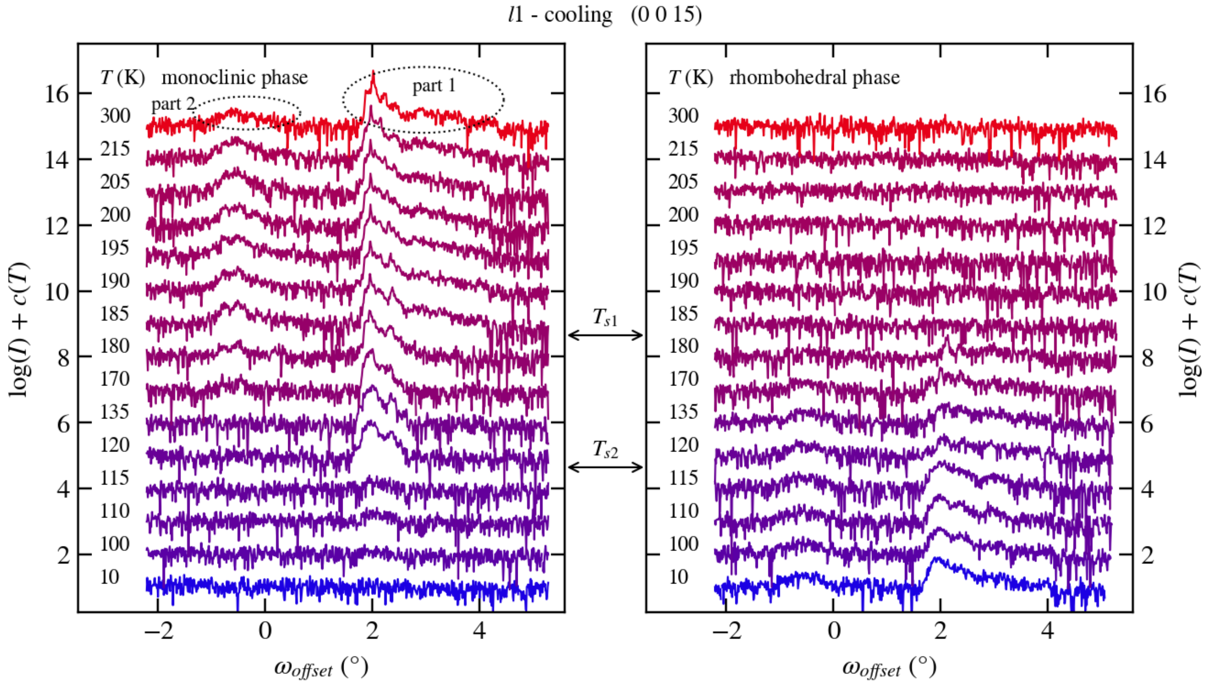

Fig. S6: ω_{offset} profile of $(0\ 0\ 15)_{hex}$ diffraction maximum. Two distinct parts (part 1 and part 2) are possible to observe with their distinct transition temperatures T_{s1} and T_{s2}. For more details see the main text.

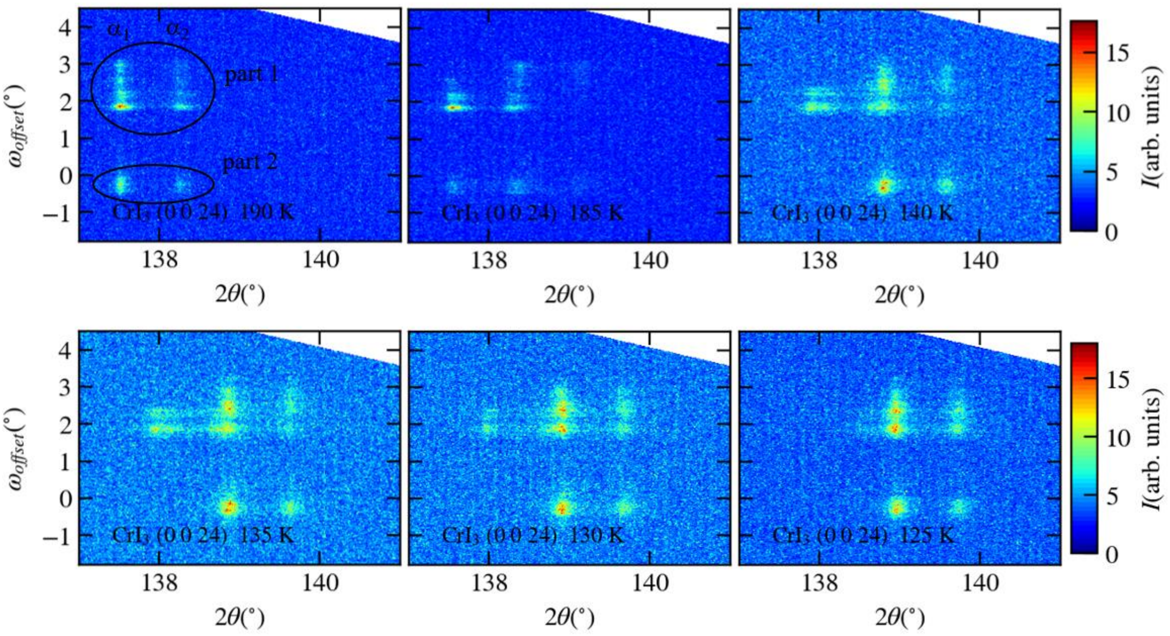

Fig. S7: ω_{offset}-2θ maps of $(0\ 0\ 24)_{hex}$ diffraction maximum showing the temperature dependence through the structural transition in CrI_3 during cooling l_2. It has to be noted that the $Cu_{K\alpha1,2}$ radiation was used and the diffraction peaks are doublets, see labels α_1 and α_2.

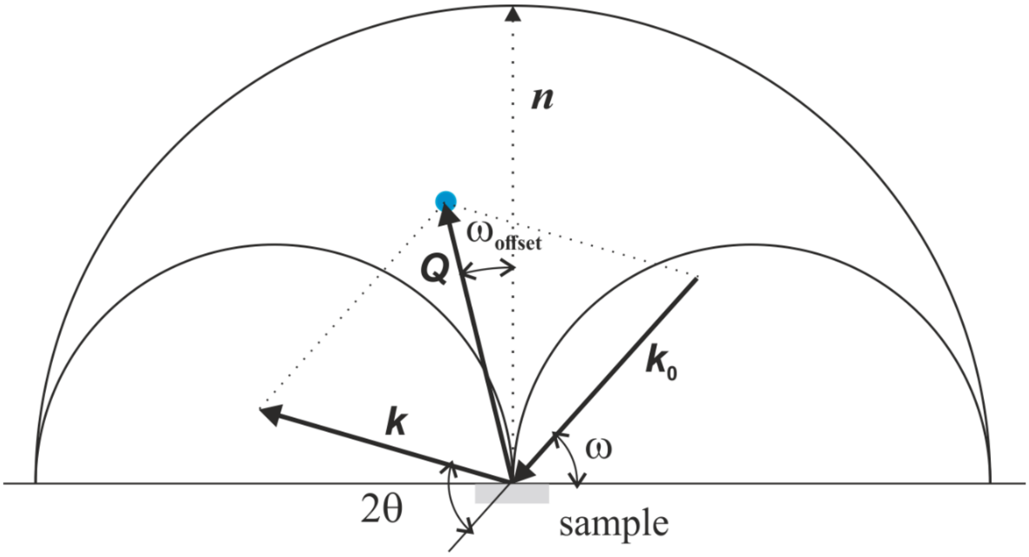

Fig. S8: Graphical representation of angles ω, 2θ and ω_{offset} corresponding to angle of incidence, angle between incident and diffracted beam and angle between diffraction vector $\boldsymbol{Q}$ and normal $\boldsymbol{n}$ of the sample surface respectively. The $\boldsymbol{k_0}$ and $\boldsymbol{k}$ are wave vectors of incident and diffracted beams.

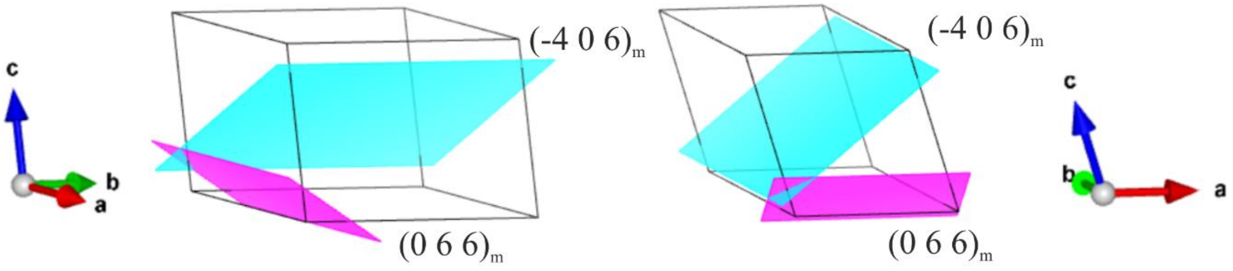

Fig. S9: Orientation of (0 6 6)$_m$ and (-4 0 6)$_m$ lattice planes within the monoclinic unit cell. The figures were plotted using the VESTA software[5].

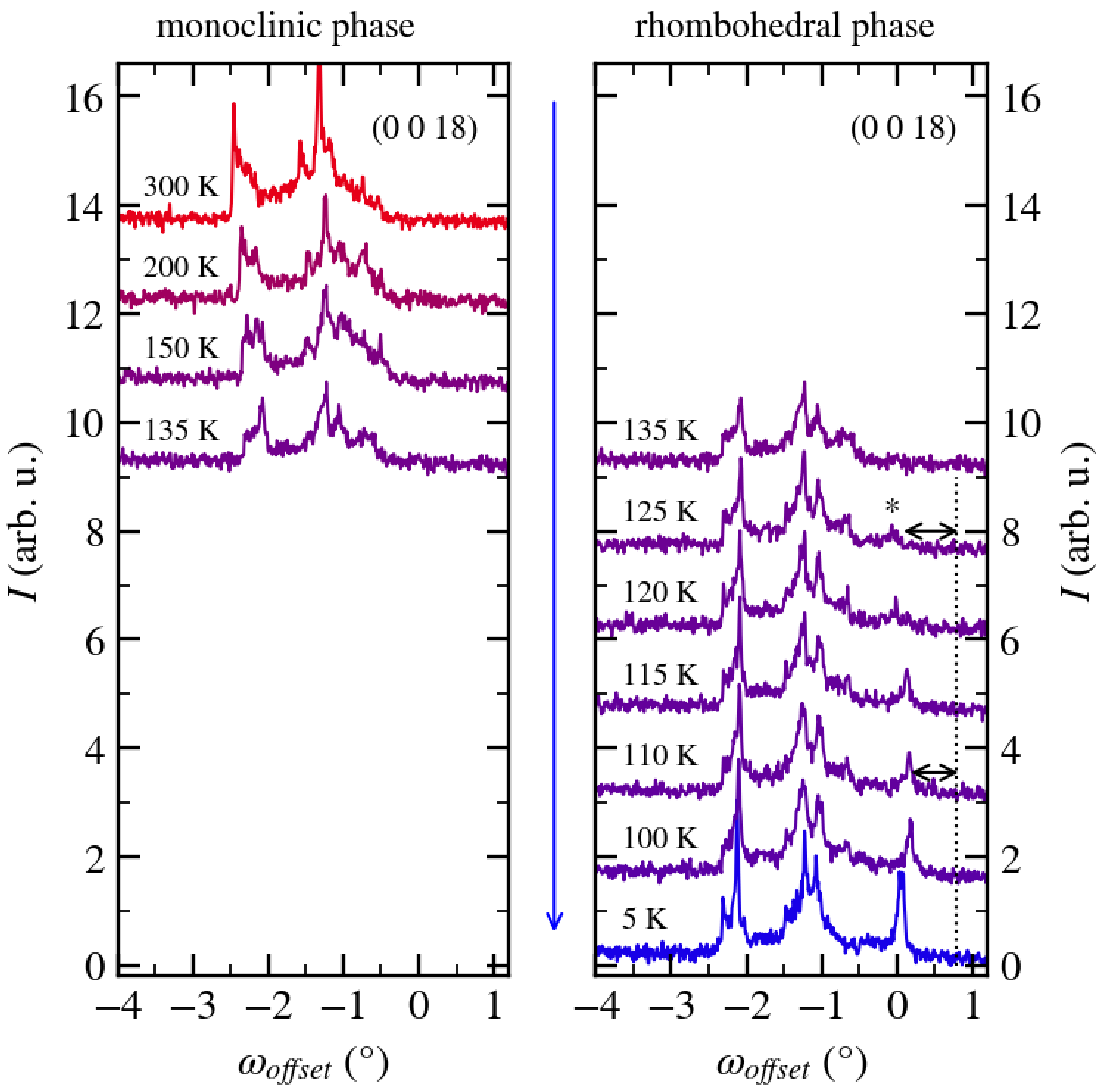

Fig. S10: ω_{offset} profile of (0 0 18) diffraction showing the formation of new mosaic block after the transition around 135 K. It has to be stressed that this sample is different from the other two samples used in this study and was not used during the data analysis. This sample contains at least three twins which would make the data analysis very complicated. But still can be used as evidence for the formation of a new mosaic block during the transition marked by a star at the right panel.

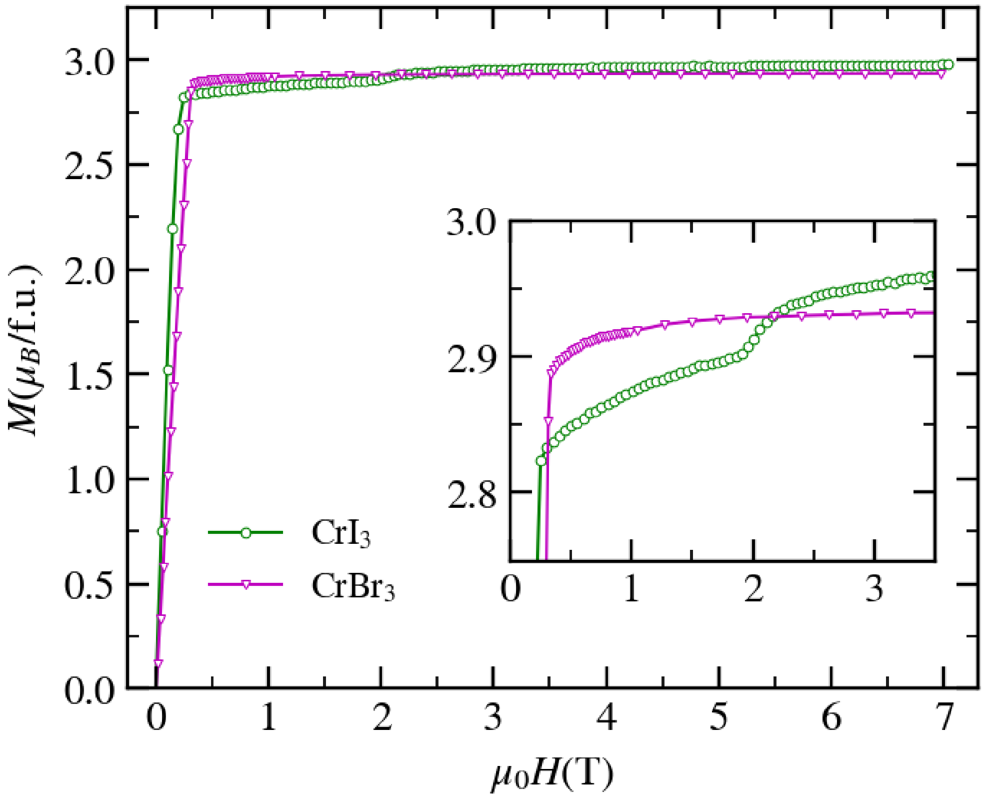

Fig. S11: Comparison of magnetization curves of CrI_3 and $CrBr_3$. The antiferromagnetic contribution in CrI_3 (related to the stacking faults) is missing in $CrBr_3$.

T **(K)**	***a*** **(Å)**	***b*** **(Å)**	***c*****(Å)**	***α*** **(°)**	***β*** **(°)**	***γ*** **(°)**
300	6.865(1)	11.888(2)	6.991(1)	90	108.480(2)	90
250	6.859(1)	11.878(2)	6.982(1)	90	108.484(2)	90
200	6.854(1)	11.870(2)	6.973(1)	90	108.491(3)	90
190	6.852(1)	11.868(2)	6.971(1)	90	108.492(3)	90
185	6.844(1)	6.844(1)	19.776(1)	90	90	120
170	6.843(1)	6.843(1)	19.767(1)	90	90	120
150	6.841(1)	6.841(1)	19.754(1)	90	90	120
130	6.840(1)	6.840(1)	19.742(1)	90	90	120
100	6.838(1)	6.838(1)	19.723(1)	90	90	120
70	6.837(1)	6.837(1)	19.703(1)	90	90	120
60	6.836(1)	6.836(1)	19.696(1)	90	90	120
50	6.836(1)	6.836(1)	19.687(1)	90	90	120
40	6.835(1)	6.835(1)	19.680(1)	90	90	120
30	6.835(1)	6.835(1)	19.675(1)	90	90	120
20	6.835(1)	6.835(1)	19.671(1)	90	90	120
10	6.834(1)	6.834(1)	19.669(1)	90	90	120
5	6.834(1)	6.834(1)	19.669(1)	90	90	120

Tab. S1: Lattice parameters measured during cooling at selected temperature points. We note that the error bars in Fig. 2 show the error connected with the accuracy (σ_c = 0.008 Å, $\sigma_{a,b}$ = 0.01 Å, σ_β = 0.01°).

REFERENCES

1. McGuire, M. A.; Dixit, H.; Cooper, V. R.; Sales, B. C., Coupling of Crystal Structure and Magnetism in the Layered, Ferromagnetic Insulator CrI3. *Chemistry of Materials* **2015,** *27* (2), 612-620.

2. Dolezal, P.; Kratochvilova, M.; Holy, V.; Cermak, P.; Sechovsky, V.; Dusek, M.; Misek, M.; Chakraborty, T.; Noda, Y.; Son, S.; Park, J. G., Crystal structures and phase transitions of the van der Waals ferromagnet VI3. *Physical Review Materials* **2019,** *3* (12), 121401.

3. Kratochvílová, M.; Doležal, P.; Hovančík, D.; Pospíšil, J.; Bendová, A.; Dušek, M.; Holý, V.; Sechovský, V., Crystal structure evolution in the van der Waals vanadium trihalides. Journal of Physics-Condensed Matter **2022**, 34 (29) 294007.

4. Kong, T.; Guo, S.; Ni, D. R.; Cava, R. J., Crystal structure and magnetic properties of the layered van der Waals compound VBr3. *Physical Review Materials* **2019**, 3 (8), 084419.

5. K. Momma and F. Izumi, VESTA 3 for three-dimensional visualization of crystal, volumetric and morphology data, J. Appl. Crystallogr., **2011**, (44) 1272-1276.
